# Supplementary material for: Cardiac Autonomic Dysfunction Is Associated with Severity of REM Sleep without Atonia in Isolated REM Sleep Behavior Disorder
Source: J Clin Med. 2021 Nov 19;10(22):5414. doi: 10.3390/jcm10225414 (PMC8621819; doi:10.3390/jcm10225414)
Supplement: Supplementary file 1 [file jcm-10-05414-s001.zip › jcm-1462263-supplementary.pdf]

Table S1. Results of PSG data in patients with iRBD who performed MIBG scan.

PSG data	Patients with iRBD/MIBG scan done (n=39)
Total sleep time (min)	380.9±58.9
N1 (%)	13.6±5.9
N2 (%)	49.7±8.6
N3 (%)	16.3±9.1
REM (%)	21.0±7.3
Latency to sleep onset	17.3±32.6
Latency to sleep stage 2 (min)	23.0±33.4
Latency to REM sleep stage (min)	117.4±70.1
RWA (%)	26.9±22.5
Sleep efficacy (%)	78.5±11.5
Apnea-Hypopnea index, Total	4.2±6.3
Arousal index, Total	12.2±7.6
PLMS index, Total	13.9±22.4

Data are mean±SD.

RWA: REM sleep Without Atonia

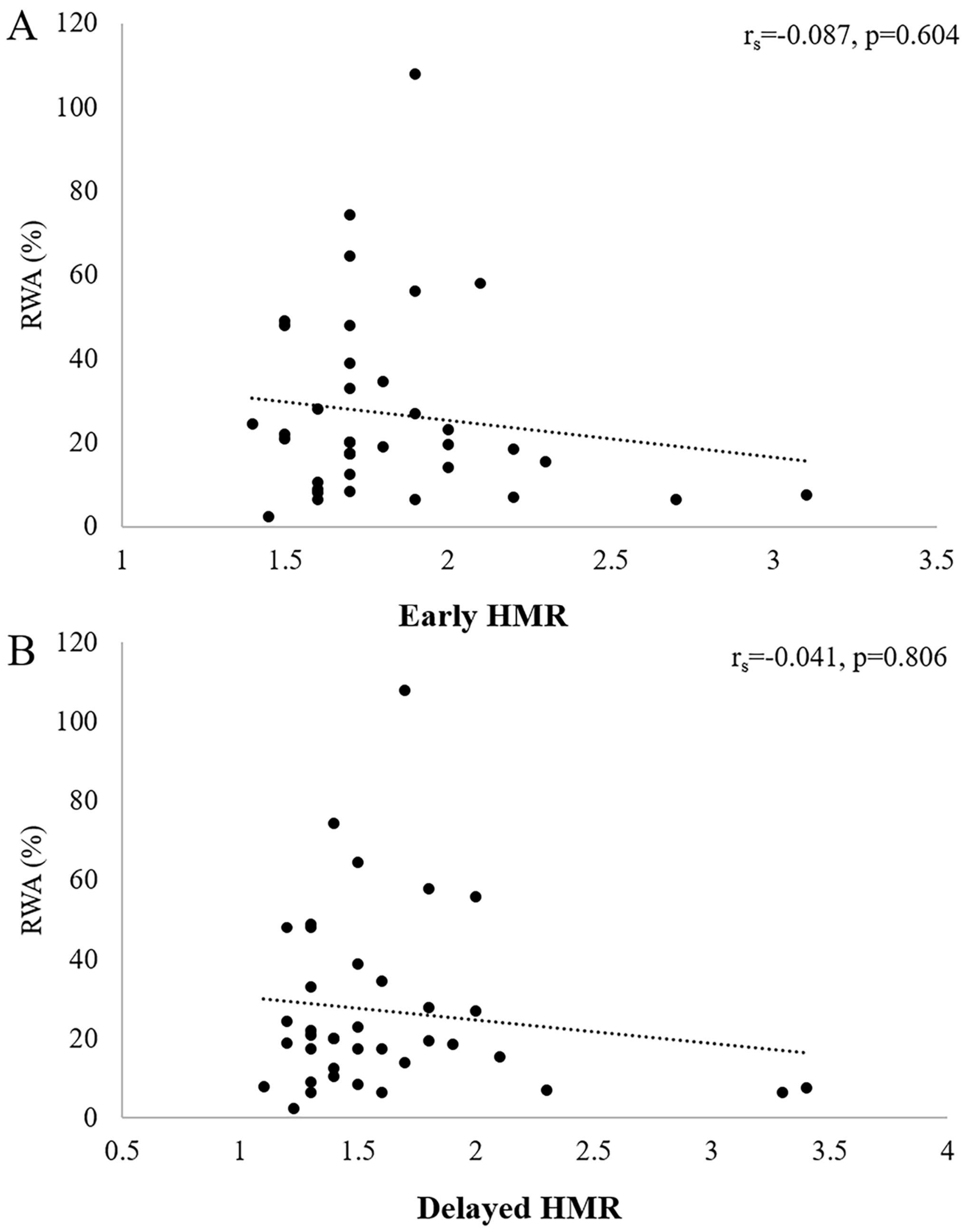

Figure S1. Correlation analysis between RWA (%) and early (A) and delayed (B) HMR of MIBG myocardial scintigraphy.
